# Almost Time

**Published:** 1891-06

**Authors:** 


### ALMOST TIME!

Almost time for the pretty white daisies
Out of their sleep to awaken at last,
And over the meadows, with grasses and clover,
To bud and to blossom, and grow so fast.
Almost time for the buttercups yellow,
The ferns and the flowers, the roses and all,
To awaken from slumber, and merrily hasten
To gladden our hearts at the spring's first call.

Almost time for the skies to grow bluer,
And breezes to soften, and days to grow long ;
For eyes to grow brighter, and hearts to grow gladder,
And earth to rejoice in her jubilant song.
Almost time for the sweetest of seasons ;
Nearer it comes with each new-born day,
And soon the smiles of the beautiful spring-time
Winter's cold shadows will chase away !—*Ex.*

